# Efficient generation of influenza virus with a mouse RNA polymerase I-driven all-in-one plasmid

**DOI:** 10.1186/s12985-015-0321-5

**Published:** 2015-06-22

**Authors:** Xiangmin Zhang, Roy Curtiss

**Affiliations:** Center for Infectious Diseases and Vaccinology, The Biodesign Institute, Arizona State University, Tempe, AZ 85287 USA; Department of Pharmaceutical Sciences, Eugene Applebaum College of Pharmacy/Health Sciences, Wayne State University, Detroit, MI USA; School of Life Science, Arizona State University, Tempe, AZ 85287 USA; Department of Infectious Diseases and Pathology, College of Veterinary Medicine, University of Florida, PO Box 110880, Gainesville, FL 32611-0880 USA

**Keywords:** Influenza virus, Reverse genetics, Mouse RNA polymerase I promoter

## Abstract

**Background:**

The current influenza vaccines are effective against seasonal influenza, but cannot be manufactured in a timely manner for a sudden pandemic or to be cost-effective to immunize huge flocks of birds. We propose a novel influenza vaccine composing a bacterial carrier and a plasmid cargo. In the immunized subjects, the bacterial carrier invades and releases its cargo into host cells where the plasmid expresses viral RNAs and proteins for reconstitution of attenuated influenza virus. Here we aimed to construct a mouse PolI-driven plasmid for efficient production of influenza virus.

**Results:**

A plasmid was constructed to express all influenza viral RNAs and proteins. This all-in-one plasmid resulted in 10^5^–10^6^ 50 % tissue culture infective dose (TCID_50_)/mL of influenza A virus in baby hamster kidney (BHK-21) cells on the third day post-transfection, and also reconstituted influenza virus in Madin–Darby canine kidney (MDCK) and Chinese hamster ovary (CHO) cells. A 6-unit plasmid was constructed by deleting the HA and NA cassettes from the all-in-one plasmid. Cotransfection of BHK-21 cells with the 6-unit plasmid and the two other plasmids encoding the HA or NA genes resulted in influenza virus titers similar to those produced by the 1-plasmid method.

**Conclusions:**

An all-in-one plasmid and a 3-plasmid murine PolI-driven reverse genetics systems were developed, and efficiently reconstituted influenza virus in BHK-21 cells. The all-in-one plasmid may serve as a tool to determine the factors inhibiting virus generation from a large size plasmid. In addition, we recommend a simple and robust “1 + 2” approach to generate influenza vaccine seed virus.

## Background

Influenza viruses belong to the *Orthomyxoviridae* family, which are characterized by segmented negative sense RNA genomes. The viral RNAs (vRNAs) are bound by nucleoprotein (NP) and three viral polymerase subunits (PB1, PB2, and PA) to form a ribonucleoprotein (RNP) complex, the minimal replication unit [1–3]. Influenza vRNAs have conserved 5′ and 3′ terminals that contain all the signals necessary for transcription, replication, and packaging [4, 5]. vRNAs with precise 5′ and 3′ ends are generally obtained using the RNA polymerase I (PolI) promoter [6, 7]. Viral polymerase subunits and nucleoprotein are synthesized under the regulation of the RNA polymerase II (PolII) promoter [8]. To generate influenza virus, pure plasmid-based reverse genetics systems are developed using 12–17 plasmids [9, 10]. Invention of the PolI-PolII bidirectional transcription vector results in a more robust 8-plasmid system [11, 12]. Due to the fact that PolI promoters are species-specific [13], different reverse genetics systems are developed for primate, avian, and canine cells [9, 14–16]. A universal T7 promoter-based system has been built, but shows less efficiency than the PolI-based bidirectional transcription system [17]. Through tandem ligation of multiple viral gene cassettes, influenza virus is reconstituted using a 3-plasmid system [18], and a single DNA construct [19, 20].

An important application of reverse genetics is the generation of influenza vaccine seed viruses [21–23]. Usually, seasonal influenza vaccine is composed of two influenza A viruses and one influenza B virus. Using the reverse genetics method, each seed virus is generated with HA and NA from a circulating strain, and the remaining six segments from a high-yield strain or an attenuated strain [24–26]. Nonetheless, manufacture of the vaccines still heavily relies on embryonated hens’ eggs or cell culture to grow the viruses [27], two time-consuming processes which are unlikely to provide adequate vaccines for a sudden influenza pandemic. The majority of vaccines are administered through intramuscular injection or by individually nasal spray, two labor-intensive ways which further increase the cost of current vaccines in vaccinating huge flocks of birds. To develop a quick-manufacturable and low-cost influenza vaccine, we proposed that an influenza viral genome-encoding plasmid could be delivered *in vivo* by a bacterial carrier, resulting in production of attenuated influenza viruses to induce protective immunity against influenza [19]. In a feasibility study, we constructed a chicken PolI-driven plasmid expressing all vRNAs, NP and polymerase from a influenza A virus (A/WSN/33) [19]. Generation of influenza virus was found in cultured avian cells infected by auxotrophic S*almonella* strains carrying the plasmid or its derivatives, but not in chickens inoculated with the recombinant *Salmonella* [28]. The study proved that a single plasmid could be constructed to reconstitute influenza virus and stably maintained in engineered *Salmonella* strains. The engineered *Salmonella* could deliver the plasmid into host cells resulting in generation of live influenza virus. Nonetheless, to make the hypothesized influenza vaccine, the one-plasmid reverse genetics system still needs to be improved in virus generation efficiency, and the *Salmonella* carrier has to be engineered to deliver plasmid efficiently, especially *in vivo*.

The mouse represents an easily handling animal and is widely used in studies of influenza virus, *Salmonella* and vaccine development. A large number of well-characterized rodent cell lines provide another advantage for *in vitro* study. Therefore, a mouse PolI-driven one-plasmid system is very useful to determine the factors limiting virus generation and *Salmonella*-mediated plasmid delivery. Some rodent cell lines, such as Chinese hamster ovary (CHO) cells and baby hamster kidney (BHK-21) cells, are widely used for research and vaccine development [29–31]. BHK-21 cells synthesize α-2, 3 and α-2, 6 sialic acid (SA) linked receptors [32], and support the growth of influenza A and B viruses [32, 33]. BHK-21 cells are also highly transfectable, even with 30-kb plasmids [34], and therefore are an appropriate cell substrate for rodent PolI-based reverse genetics systems.

In this study, we constructed a mouse PolI-driven all-in-one plasmid to reconstitute influenza virus. A “1 + 2” plasmid strategy was also tested to simplify the yearly generation of influenza vaccine seeds.

## Results

### Plasmid construction

**Fig. 1:**
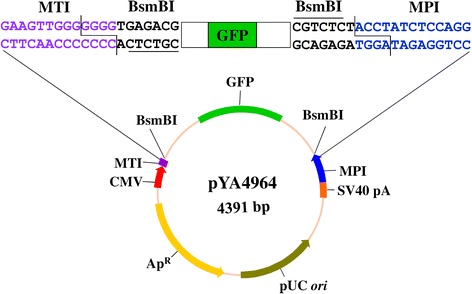
Illustration of the bidirectional vector. A cytomegalovirus (CMV) promoter and Simian virus 40 (SV40) polyadenylation signal (pA) direct the synthesis of mRNA. The mouse RNA PolI promoter (MPI) and terminator (MTI) compose the vRNA transcription unit. Between the two BsmBI sites, there is a prokaryotic green fluorescent protein (GFP) gene cassette. The MPI and MTI sequences adjacent to GFP cassette are shown in blue or purple. Substitution of the GFP cassette with the influenza virus gene resulted in the loss of green fluorescent protein expression in the bacterial host

Using the truncated mouse PolI promoter (MPI, 250 bp), plasmid pYA4924 was constructed to transcribe negative sense EGFP RNA flanked by the 5′ and 3′ non-translating regions of influenza virus M segment (vRNA-like). EGFP expression was observed in BHK-21 cells only when pYA4924 has been cotransfected with plasmids encoding influenza polymerase and NP (Data not shown), suggesting that the cloned MPI is functional in hamster cells. By combining MPI with the SV40 polyadenylation sequence (SV40 pA, 131 bp), truncated mouse PolI terminator (MTI, 41 bp), and functional truncated CMV promoter (188 bp), a bidirectional CMV/MPI-driven transcription vector, pYA4963, was constructed. Two BsmBI sites were designed between MPI and MTI to allow precise insertion of influenza cDNA. A BamHI site was designed between the two BsmBI sites. By inserting a prokaryotic GFP-expression cassette at the BamHI site, an easy-to-use bidirectional vector pYA4964 was constructed (Fig. 1). Substitution of the GFP cassette with influenza cDNA resulted in loss of green fluorescence in bacterial host cells. The GFP-based selection method facilitated construction of the mouse PolI-driven 8-plasmid system that included plasmids pYA4965 (PB1), pYA4966 (PB2), pYA4967 (PA), pYA4968 (NP), pYA4969 (HA), pYA4970 (NA), pYA4971 (M), and pYA4972 (NS) (Table 1).

**Table 1:** Plasmids used in the study

Plasmid type	Plasmid (related properties)	*ori* and marker
Vector plasmid	pcDNA3.1(−) (CMV, SV40 pA)	pUC *ori*, Ap^R^
T-vector precursor	pYA4518 (AhdI-GFP cassette-AhdI)	p15A *ori*, Cm^R^
Reporter plasmid	pYA4392 (CPI-EGFP), pYA4924 (MPI-EGFP)	
	pYA4332 (GFP cassette)	pUC*ori*, Ap^R^
	pYA4731 (CMV-mCherry)	
	pYA4732 (CMV-mCherry, PB2, PB1, PA, NP, HA, NA, M, NS)	p15A *ori*, Cm^R^;
MPI-based bidirectional vector	pYA4963 (CMV-MPI), pYA4964 (CMV-GFP cassette-MPI)	pUC*ori*, Ap^R^
CPI-based 8-plasmid system	pYA4383 (PB2), pYA4384 (PB1), pYA4385 (PA), pYA4386 (NP), pYA4388 (HA), pYA4389 (NA), pYA4390 (M), pYA4391 (NS)	
MPI-based 8-plasmid system	pYA4965 (PB1), pYA4966 (PB2), pYA4967 (PA), pYA4968 (NP), pYA4969 (HA), pYA4970 (NA), pYA4971 (M), pYA4972 (NS)	
1-unit plasmid	pYA4973 (PB1), pYA4974 (NP), pYA4975 (NA), pYA4976 (NS); pYA4977 (PB1,GFP), pYA4978 (NP, GFP), pYA4979 (NA,GFP), pYA4980 (NS,GFP)	p15A *ori*, Cm^R^
2-unit plasmid	pYA4981 (PB1,PB2), pYA4982 (NP,PA), pYA4983 (NA,HA), pYA4984 (NS,M), pYA4985 (PB1,PB2,GFP), pYA4986 (NP,PA,GFP), pYA4987 (NS,M,GFP)	
4-unit plasmid	pYA4988 (NP, PA, PB1, PB2,GFP), pYA4989 (NS, M, NA, HA)	
8-unit plasmid	pYA4990 (NP, PA, PB1, PB2, NS, M, NA, HA)	
6-unit plasmid	pYA5000 (NP, PA, PB1, PB2, NS, M)	

**Fig. 2:**
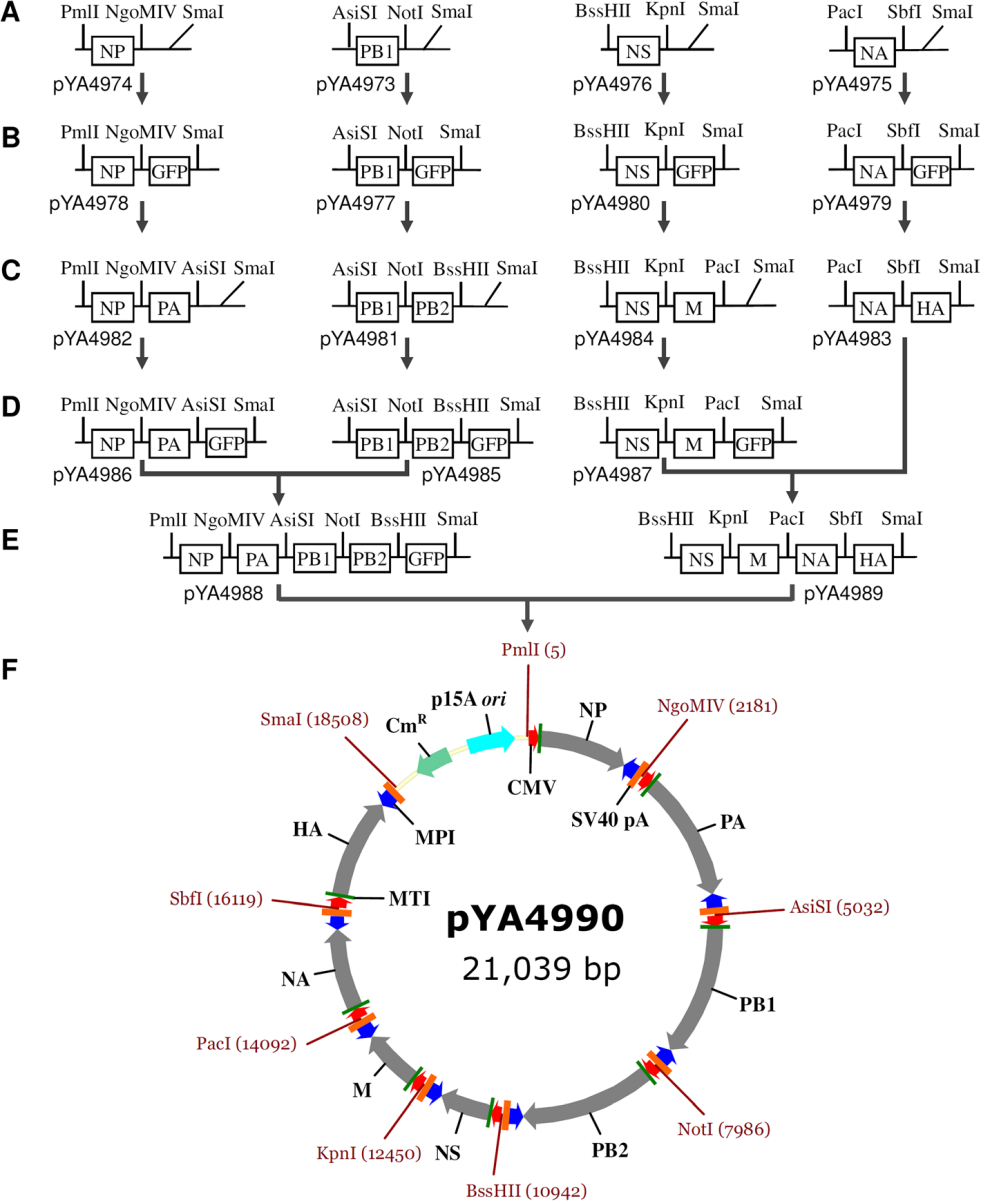
Construction of the all-in-one plasmid. **a** Using primers engineered with unique restriction enzyme cleavage sites, the NP, PB1, NS, and NA cassettes from pYA4968 (NP), pYA4965 (PB1), pYA4972 (NS), and pYA4970 (NA) were amplified and cloned into the pYA4518-derived T-vector to obtain four 1-unit plasmids pYA4974 (NP), pYA4973 (PB1), pYA4976(NS), and pYA4975 (NA). **b** The GFP cassettes were amplified from pYA4964 and inserted into the four 1-unit plasmids between SmaI, and the adjacent enzyme site downstream each influenza gene cassette. The resulting plasmids were pYA4978 (NP, GFP), pYA4977 (PB1, GFP), pYA4980 (NS, GFP), and pYA4979 (NA, GFP). **c** The PA, PB2, M, and HA cassettes were amplified from pYA4967 (PA), pYA4966 (PB2), pYA4971 (M), and pYA4969 (HA) to replace the GFP cassettes in the 1-unit plasmids from step b. The resulting 2-unit plasmids were pYA4982 (NP, PA), pYA4981 (PB1, PB2), pYA4984 (NS, M), and pYA4983 (NA, HA). **d** The GFP cassettes were amplified from pYA4964 and inserted into three of the 2-unit plasmids. The resulting plasmids were pYA4986 (NP, PA, GFP), pYA4985 (PB1, PB2, GFP), and pYA4987 (NS, M, GFP). **e** The AsiSI-XmaI fragments from plasmids pYA4986 (NP, PA, GFP) and pYA4985 (PB1, PB2, GFP) were combined to obtain a 4-unit plasmid pYA4988 (NP, PA, PB1, PB2, GFP). The AsiSI-XmaI fragments from plasmids pYA4987 (NS, M, GFP) and pYA4983 (NA, HA) were combined to obtain another 4-unit plasmid pYA4989 (NS, M, NA, HA). **f** The two 4-unit plasmids were digested with BssHII and XmaI. Combination of the fragments containing influenza cassettes resulted in the all-in-one plasmid pYA4990. The PA, PB2, M, HA, and GFP cassettes had triple C (CCC) at their 3′-end for connecting to the SmaI-generated blunt end (as shown by primer sequence in Table 3). The SmaI and XmaI endonucleases were isoschizomers. For clarity, each element is labeled at only one location in plasmid pYA4990

To combine all CMV/MPI-driven influenza gene cassettes on one plasmid, four influenza cassettes were first cloned into a linearized vector which has single 3′-T overhangs on both ends (T-vector) (Fig. 2). Unique restriction enzyme cleavage sites were designed at each end of the cassette to allow insertion of the other cassette. A prokaryotic GFP cassette was first inserted between two adjacent enzyme sites, followed by selection of green colonies under excitation with 488 nm light. The GFP cassettes were then substituted with influenza cassettes by selecting non-fluorescent colonies. These steps resulted in four 2-unit plasmids, with each unit representing a bidirectional influenza cassette. The GFP cassettes were again inserted into three of the 2-unit plasmids. Each of the two 2-unit plasmids were combined to obtain two 4-unit plasmids, pYA4988 and pYA4989. Combination of the 4-unit plasmids resulted in the all-in-one plasmid pYA4990 which was further confirmed by sequencing. The all-in-one plasmid was designed to transcribe all eight vRNAs and eight mRNAs (10 mRNAs after spliced) in rodent cells. The HA and NA cassettes in pYA4990 were deleted to form a 6-unit plasmid that composes a “1 + 2” plasmid system with the HA and NA plasmids (pYA4969 and pYA4970).

### Plasmid stability in *E. coli*

**Fig. 3:**
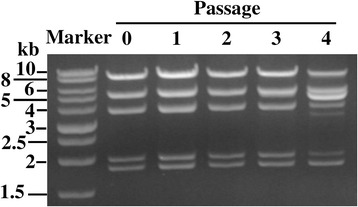
Homologous recombination of plasmid pYA4990. *E. coli recA1* mutant strain EPI300 was transformed with plasmid pYA4990. A single colony was rotary cultured in LB media containing 25 μg/ml chloramphenicol at 37 °C for 12 h (passage 0). The culture was diluted at the ratio of 1:1000, and cultured at same condition for additional four passages. Plasmid DNA was extracted from each passage, and digested with EcoRI and KpnI. From an intact plasmid pYA4990, the size of five theoretical fragments are 8384 bp, 5166 bp, 3747 bp, 1944 bp, and 1798 bp

The all-in-one plasmid contains repetitive sequences in the promoter and terminator elements flanking each of the viral genes (Fig. 2). Homologous DNA recombination among these repetitive sequences impairs plasmid stability. To stably maintain and amplify the plasmid, an *E. coli* strain with a *recA* mutation was used. As shown in Fig. 3, plasmid pYA4990 was electroporated into *E. coli* strain EPI300 (RecA^−^). A single colony was first inoculated in 3 ml of growth media, and passaged at dilution ratio of 1:1000. DNA recombination was monitored by restriction enzyme digestion of plasmid DNA isolated from different passages. At the fourth passage, there was apparent change in the restriction map of pYA4990, indicating significantly impaired plasmid structure. Qualitative evaluation of the DNA bands on agarose gels suggested that the plasmid structure was stable for the first 3 passages in *E. coli* strain EPI300. From three independent assays, we observed consistent results. We also tested pYA4490 recombination in another *E. coli* strain Stbl3 (RecA^−^) and got similar result (data not shown). In theory, three passages could result in 3 × 10^9^ ml of bacterial culture for plasmid preparation. Therefore, DNA recombination does not represent a big problem with the all-in-one plasmid when an *E. coli* strain with a *recA* mutation is used during cloning and plasmid amplification processes.

### Virus generation

To test virus generation, 4 μg of the all-in-one plasmid pYA4990 was used to transfect BHK-21, CHO, or MDCK cells. For BHK-21 cells, the virus titers ranged from 1 to 3 × 10^5^ median tissue culture infective dose (TCID_50_) per mL on the third day post-transfection (Experiments 1–3; Table 2). When less confluent BHK-21 cells were used in transfection, the virus titers went up to 3–6 × 10^6^ TCID_50_/mL (Experiments 4–6). In contrast, the CHO cells showed very poor virus generation (0–10 TCID_50_/mL) on the third day post-transfection. The MDCK cells showed inconsistent virus generation ranged from 0 to 1 × 10^6^ TCID_50_/mL on the third day post-transfection. In Experiments 4–6, the virus titers went up to 1–3 × 10^7^ TCID_50_/mL on the fifth day (not shown in Table 2). As control, 4 μg of the 6-unit plasmid pYA5000 was transfected into each type of cells. No virus generation was observed. To test the “1 + 2” plasmid system, 4 μg of the 6-unit plasmid pYA5000 was cotransfected into either BHK-21 or MDCK cells with 2 μg of the HA plasmid pYA4969 and 2 μg of the NA plasmid pYA4970. Although, no virus generation was observed in MDCK cells, the “1 + 2” approach resulted in similar titers of influenza virus in BHK-21 cells as the all-in-one plasmid pYA4990 in the parallel experiments (Table 2).

**Table 2:** Influenza virus generation in different cell lines (TCID_50_/mL)

Cell	Plasmid	Transfection					
		1	2	3	4	5	6
BHK-21	“1 + 2” plasmids^a^	3 × 10^5^	1 × 10^5^	1 × 10^5^	-	-	-
	pYA4990	1 × 10^5^	3 × 10^5^	3 × 10^5^	6 × 10^6^	3 × 10^6^	6 × 10^6^
	pYA5000	0	-	-	-	-	-
CHO	pYA4990	10	0	10	-	-	-
	pYA5000	0	-	-	-	-	-
MDCK	“1 + 2” plasmids	0	0	0	-	-	-
	pYA4990	3 × 10^5^	3 × 10^5^	0	3 × 10^3^	1 × 10^6^	3 × 10^3^
	pYA5000	0	-	-	-	-	-

- Not performed

^a^ pYA4969, pYA4970, and pYA5000

We found that the transfected BHK-21 cells detached but remained viable in media containing 2 μg/ml TPCK-trypsin, the virus yield was around 10^5^ TCID50/ml (data not shown). TPCK-trypsin at a concentration of 0.2 μg/ml did not apparently affect cell attachment but provided as consistent virus yields as the trypsin-free condition. The generated viruses were confirmed to be influenza A virus by western blot analysis with antibodies specific to the NP and M2 proteins (data not shown). We also extracted vRNA from influenza virus generated in BHK-21 cells. The HA and NA segments were reverse transcribed and amplified by PCR. Sequencing results showed that influenza virus from three experiments had identical HA and NA coding sequences as encoded by the all-in-one plasmid.

### Transfection efficiency

**Fig. 4:**
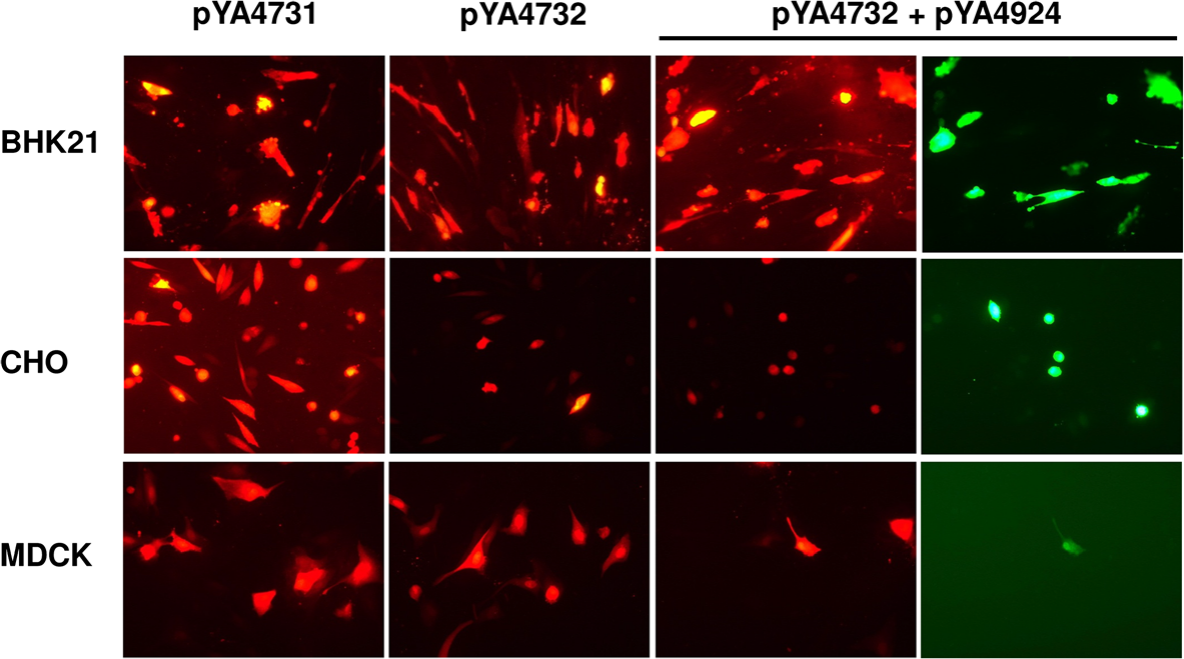
Analysis of gene expression in BHK-21, CHO, and MDCK cells. The cells were transfected with reporter plasmids carrying a CMV-driven mCherry cassette. The large pYA4732 plasmid showed significantly lower mCherry expression in CHO cells than the small pYA4731 plasmid. Cells were also cotransfected with pYA4732 and pYA4924. The EGFP expression indicated that vRNA-like EGFP RNA was generated from pYA4924 and converted into mRNA for EGFP synthesis in the presence of the influenza NP and polymerase provided by pYA4732. Compared with BHK-21 and CHO cells, very few MDCK cells expressed EGFP. Expression of EGFP and mCherry were recorded from the same field

The BHK-21, CHO, and MDCK cells in 80–90 % confluence were transfected with a 6.1-kb plasmid pYA4731 (CMV-mCherry) or a 25.3-kb plasmid pYA4732 (CMV-mCherry) [19]. Both plasmids resulted in similar levels of mCherry expression in BHK-21 and in MDCK cells (Fig. 4). The CHO cells showed preferential uptake of the smaller plasmid. The three types of cells were cotransfected with pYA4924 and pYA4732 which encodes influenza viral polymerase and NP. The BHK-21 and CHO cells showed efficient EGFP expression, indicating that the vRNA-like molecules were converted into mRNA. In contrast, only numerous MDCK cells expressed EGFP, which suggests that the mouse PolI promoter is poorly active in canine cells.

## Discussion

To combat influenza virus infections, the inactivated and attenuated vaccines remain the most effective tools [35]. The time-consuming manufacture process and labor-intensive immunization methods of current vaccines render them suboptimal for an influenza pandemic or huge flocks of birds. Recently, we proposed a novel influenza vaccine which composes a bacterial carrier harboring an influenza plasmid [19, 28]. The vaccine can be quickly propagated in simple media in large scale, and potentially be orally administered. *In vivo* delivery of the influenza plasmid results in generation of attenuated influenza virus which in turn induces protective immunity. The feasibility study showed that such a form of recombinant bacteria could be constructed and used to mediate generation of influenza virus [28]. As expected, there are limiting factors still needing to be overcome, such as inefficient virus generation from the influenza plasmid. In this study we aimed to construct a mouse PolI-driven plasmid for reconstitution of influenza virus, so that we can determine the factors limiting virus generation in the future using well-characterized rodent cell lines.

The whole set of vRNA, neucleoprotein and three polymerase subunits are the minimal components required to initiate influenza virus replication and packaging [3, 9]. The virus production efficiency may be improved by coexpression of proteins from M, MS, HA and NA segments [9]. Synthesis of these proteins requires four more copies of PolII promoter and terminator sequence. This increase in plasmid size, however, has a negative effect on the nuclear import of plasmid DNA and virus production [19, 36]. To solve this problem, we used a short PolII terminator (SV40 pA) and a truncated less-active CMV promoter (data not shown). With these changes, we made the 21-kb all-in-one plasmid, which is 2.6 kb less in size than the 8-cassette influenza plasmid described previously [19]. We did not consider to use the T7 promoter-based system, as it is less efficient than the PolI-PolII bidirectional transcription system [17], and requires coexpression of T7 RNA polymerase.

Transfection of BHK-21 cells with plasmid pYA4990 resulted in high titers of influenza virus, most likely because the cells synthesize α-2, 6 SA-linked influenza receptor [32] and can be efficiently transfected by large size plasmids [34]. The CHO cells, another hamster cell line, only yielded merely detectable influenza virus. One reason is that CHO cells do not have α-2, 6 SA-linked receptor to support influenza virus propagation [37, 38]. These two hamster cell lines may help determine the mechanism limiting nuclear import of the large size plasmid and the factors inhibiting virus generation. A plasmid enable to overcome the barriers should reconstitute influenza virus efficiently in wide range of cells such as intestinal epithelial cells, therefore can be used to develop the proposed influenza vaccine.

Each seed virus of influenza vaccines is generated yearly with HA and NA genes from a circulating influenza virus, and the remaining backbone segments (PB1, PB2, PA, NP, M and NS) from a high-yield or cold-adapted virus [26, 39, 40]. Since the backbone encoding segments remain the same every year, they can be used to make a 6-cassete plasmid. The 6-cassete plasmid forms a 3-plasmid reverse genetics system with the two plasmids encoding HA and NA genes. This “1 + 2” approach was proven successful in the BHK-21 cells (Table 2). We then tested virus generation in CHO cells which have been used to produce human vaccines and other therapeutics for many years [31]. The all-in-one plasmid could reconstitute low titers of influenza virus in CHO cells, but the “1 + 2” strategy was not successful. This may be overcome by using CHO cells expressing influenza receptor [41–43], or coculture of CHO and MDCK cells. On the other hand, it also suggests that a more comprehensive system yields higher titers of virus [18]. The 3-plasmid system should reconstituteseed virus more efficiently than an 8-plasmid system in CEF, Vero cells and MDCK cells [44]. In other circumstance, if one needs to generate a large number of influenza viruses merely different in one gene, such as NP, a 7-cassette plasmid may be constructed to compose a 2-plasmid system with the NP plasmid. If just numerous influenza viruses need to be generated, it is not worth the time to construct a complicated plasmid, and an 8-plasmid system should be used.

Given the well-known fact that the PolI promoter is species-specific, the human PolI promoter is, however, highly active in MDCK cells [45]. The mouse PolI promoter was found to be active in hamster cell lines, but showed very poor or no activity in MDCK cells. The results suggest that it is an inefficient process to produce the first viral particle in MDCK cells transfected by the all-in-one plasmid as a result of poor vRNA transcription. However, once a live influenza viral particle is generated, it can replicate rapidly in MDCK cells. Like the finding with CHO cells, the 3-plasmid system did not reconstitute influenza virus in MDCK cells. This is because cotransfection of three plasmids into MDCK cells is less efficient than a plasmid, and leads to even less vRNA production in canine cells.

Construction of a plasmid containing all influenza genes driven by different promoters could be laborious and time consuming. As shown in Fig. 2, we simplified and speeded up the process with multiple strategies including parallel construction of multiple plasmids, GFP-based selection, use of a low copy plasmid vector [19], and use of *E. coli* strains with a *recA1* mutation [46, 47]. During gel purification of vector and fragment DNA, UV transillumination causes cross-linking of nucleosides and profoundly reduces transformation frequencies of ligation product, especially for large DNA constructs [48]. During construction of the 4-unit and the all-in-one plasmids, the DNA fragments were therefore prepared by staining the agarose gel with crystal violet [49] and detected under white light illumination.

## Conclusions

We developed mouse PolI-driven reverse genetics systems of influenza virus. Both the all-in-one plasmid and “1 + 2” plasmid system were efficient in generation of influenza virus in BHK-21 cells, but not in CHO cells used in the study. The all-in-one plasmid is an ideal tool to determine the mechanism limiting nuclear import of the large size plasmid and the factors inhibiting virus generation using well-characterized rodent cell lines. In addition, we recommend a more simple and efficient “1 + 2” plasmid system to generate seed virus of influenza vaccine where one plasmid encodes backbone segments (PB1, PB2, PA, NP, M, NS) from a high-yield or cold-adapted strain, and remains the same every year. The other two plasmids are constructed yearly by cloning the HA and NA genes from a circulating strain of influenza virus.

## Methods

### Bacterial strain and cell lines

The *Escherichia coli recA1* mutant strain EPI300 (Epicentre) was used for all DNA cloning. Baby hamster kidney (BHK-21) cells, Chinese hamster ovary (CHO) cells, and Madin-Darby canine kidney (MDCK) cells were maintained in Dulbecco’s modified Eagle’s medium (DMEM) supplemented with 10 % fetal bovine serum (FBS), 100 U/mL penicillin, and 100 μg/mL streptomycin.

### Plasmid construction

The PfuUltra High-Fidelity DNA polymerase (Stratagene) was used to amplify the influenza genes and cassettes in the current study. Agarose gel containing 10 μg/mL of crystal violet was used to isolate large DNA fragments. The plasmids and primers used in the study are presented in Tables 1 and 3.

**Table 3:** Primers used in the study

Target/template	Primer	Sequence (5′ → 3′)^a^
MTI-EGFP/pYA4392	P1	ttgtcgcccggagtactggtcg
	P2	attggacctggagataggtagtagaaacaaggtag
MPI/mouse genomic DNA	P3	ctaccttgtttctactacctatctccaggtccaat
	P4	tacgtctgaggccgagggaaagc
SV40 pA/pcDNA3.1(−)	P5	tacagacatgataagatacat
	P6	cctcggcctcagacgtaaacttgtttattgcagc
CMV/pcDNA3.1(−)	P7	caacttcggaggtcgaccagtactccgggcgacaagagctctgcttatatag
	P8	ttaagatctgtacatcaatgggcgtgg
MPI/pYA4924	P9	gctgcaataaacaagtttacgtctgaggccgagg
	P10	tggtcgacctccgaagttgggggggtgagacggatccgtctctacctatctccaggtcc
GFP cassette/pYA4332	BglII-lpp	taaagatctttgttgtgtgaattaat
	BglII-5ST1T2	ttaagatcttccattattgaagcatt
PB1/pYA4384	AarI-1 PB1	taacacctgcagtcaggtagtagaaacaaggcatt
	AarI-2 PB1	ttacacctgcgactggggagcgaaagcaggcaaac
PB2/pYA4383	AarI-1 PB2	taacacctgcagtcaggtagtagaaacaaggtcgt
	AarI-2 PB2	ttacacctgcgactggggagcgaaagcaggtcaat
PA/pYA4385	BsmBI-1PA	taacgtctctaggtagtagaaacaaggtact
	BsmBI-2PA	ttacgtctctggggagcgaaagcaggtactg
NP/pYA4386	BsmBI-1NP	taacgtctctaggtagtagaaacaagggtat
	BsmBI-2NP	ttacgtctctggggagcaaaagcagggtaga
HA/pYA4388	BsmBI-1HA	taacgtctctaggtagtagaaacaagggtg
	BsmBI-2HA	ttacgtctctggggagcaaaagcaggggaa
NA/pYA4389	AarI-1NA	taacacctgcagtcaggtagtagaaacaaggagtt
	AarI-2NA	ttacacctgcgactggggagcgaaagcaggagttt
M/pYA4390	BsmBI-1 M	taacgtctctaggtagtagaaacaaggtagt
	BsmBI-2 M	ttacgtctctggggagcaaaagcaggtagat
NS/pYA4391	BsmBI-1NS	taacgtctctaggtagtagaaacaagggtgt
	BsmBI-2NS	ttacgtctctggggagcaaaagcagggtgac
NP cassette /pYA4968	PmlI-NP	cacgtgtacatcaatgggcgtggatagcg
	SmaI-NgoMIV-NP	cccgggatagccggcagacatgataagatacat
PA cassette/pYA4967	NgoMIV-PA	taagccggcgtacatcaatgggcgtggat
	GGG-AsiSI-PA	gggatagcgatcgcagacatgataagatacat
PB1 cassette/pYA4965	AsiSI-PB1	gcgatcgcgtacatcaatgggcgtggat
	SmaI-NotI-PB1	cccgggatagcggccgcagacatgataagatacat
PB2 cassette/pYA4966	NotI-PB2	taagcggccgcgtacatcaatgggcgtggat
	GGG-BssHII-PB2	gggatagcgcgcagacatgataagatacat
NS cassette/pYA4972	BssHII-NS	gcgcgcgtacatcaatgggcgtggatag
	SmaI-KpnI-NS	cccgggataggtaccagacatgataagatacat
M cassette/pYA4971	KpnI-M	taaggtaccgtacatcaatgggcgtggat
	GGG-PacI-M	gggatattaattaacagacatgataagatacat
NA cassette/pYA4970	PacI-NA	ttaattaagtacatcaatgggcgtggatag
	SmaI-SbfI-NA	cccgggatacctgcaggcagacatgataagatacat
HA cassette/pYA4969	SbfI-HA	taacctgcagggtacatcaatgggcgtggat
	GGG-HA	gggcagacatgataagatacattgatg
GFP cassette/pYA4964^b^	GGG-5ST1T2	gggtccattattgaagcatttatcaggg
	SbfI-lpp	taacctgcaggttgttgtgtgaattaatttgt
	KpnI-lpp	taaggtaccttgttgtgtgaattaatttgt
	NotI-lpp	taagcggccgcttgttgtgtgaattaatttgt
	NgoMIV-lpp	taagccggcttgttgtgtgaattaatttgt
	PacI-lpp	taattaattaattgttgtgtgaattaatttgt
	BssHII-lpp	taagcgcgcttgttgtgtgaattaatttgt
	AsiSI-lpp	taagcgatcgcttgttgtgtgaattaatttgt

^a^The restriction enzyme sites or the GGG (corresponding to 3′- triple C of the PA, PB2, M, HA, and GFP cassettes in Fig. 2) were underlined

^b^Primer GGG-5ST1T2 was paired with each of the other 7 primers for amplifying GFP cassette from pYA4964

From plasmid pYA4392 [19], the DNA fragment containing truncated mouse PolI terminator (MTI, 41 bp) and EGFP gene was amplified with primers P1 and P2. The 250 bp truncated mouse RNA PolI promoter (MPI) was amplified from BALB/c mouse genomic DNA with primers P3 and P4. The two amplified fragments were fused by PCR using primers P1 and P4. Purified PCR products were treated with dATP and Taq DNA polymerase to add 3′A at each end. Plasmid pYA4924 was constructed by cloning the treated PCR product into a lab-made T-vector derived from pYA4518 [50]. Plasmid pYA4518 had a p15A *ori*, a chloramphenicol-resistance marker (Cm^R^), and a prokaryotic green fluorescent protein (GFP) gene cassette flanked by two AhdI sites. Removal of the GFP cassette from plasmid pYA4518 through AhdI digestion resulted in a T-vector for cloning PCR products.

From plasmid pcDNA3.1(−) (Invitrogen), the SV40 polyadenylation sequence (SV40 pA, 131 bp) was amplified with primers P5 and P6, and the truncated CMV promoter (188 bp) was amplified with primers P7 and P8. The MPI was amplified from pYA4924 with primers P9 and P10. The 41-bp MTI was introduced by primers P7 and P10. A DNA fragment containing BglII-CMV-MTI-BsmBI-BamHI-BsmBI-MPI-SV40pA was obtained by fusing the three PCR products with primers P5 and P8. The purified PCR product was digested with BglII and ligated into pcDNA3.1(−) digested with BstZ17I and BglII to replace the DNA fragment containing the CMV promoter, the bovine growth hormone (BGH) pA, and the *neo* cassette. The resulting bidirectional vector was designated as pYA4963. To make an easy-to-use vector, the prokaryotic expression GFP cassette was amplified from pYA4332 with primers BglII-lpp and BglII-5ST1T2. The GFP cassette was digested with BglII and ligated into the pYA4963 vector that was digested with BamHI and treated with calf intestinal alkaline phosphatase (CIAP); the resulting plasmid was pYA4964 (Fig. 1). The linearized and ready-to-use pYA4964 was prepared by removing the GFP cassette with BsmBI. From the chicken PolI promoter based 8-plasmid system pYA4383 (PB2), pYA4384 (PB1), pYA4385 (PA), pYA4386 (NP), pYA4388 (HA), pYA4389 (NA), pYA4390 (M), and pYA4391 (NS) [19], the influenza A virus (A/WSN/33(H1N1)) genes were amplified by PCR and digested with BsmBI or AarI. Each treated gene was cloned individually into the linearized pYA4964. Only colonies that did not show green fluorescence under excitation of 488 nm were selected and verified by restriction digestion of the plasmid DNA. The resulting mouse RNA PolI-driven 8-plasmid system included plasmids pYA4965 (PB1), pYA4966 (PB2), pYA4967 (PA), pYA4968 (NP), pYA4969 (HA), pYA4970 (NA), pYA4971 (M), and pYA4972 (NS).

Using primers engineered with unique restriction enzyme cleavage sites, the influenza gene cassettes were amplified from the mouse PolI-driven plasmids. An all-in-one plasmid pYA4990 was constructed by combining all of the cassettes (Fig. 2). The pYA4990 was sequenced with 54 primers that target influenza genes (not shown). To construct a 6-unit plasmid without HA and NA cassettes, the pYA4990 was digested by PacI and SmaI. The large linearized fragment was treated with Klenow large fragment to form blunt ends, and self-ligated. The resulting plasmid was pYA5000, which served as the control or constituted the “1 + 2” plasmids system for generating influenza virus with plasmids pYA4969 (HA) and pYA4970 (NA).

### DNA recombination assay

DNA recombination of the all-in-one plasmid in *E. coli* was evaluated as described previously [28].

### Transfection

BHK-21, CHO, and MDCK cells grown in 6-well plates were transfected according to the manufacturer’s instructions. Briefly, 2 μL of Lipofectamine 2000 (Invitrogen) per μg plasmid DNA were individually diluted in 100 μL of Opti-MEM. After 5 min incubation at room temperature, the diluted transfection reagent was mixed with the DNA. After 40 min incubation at room temperature, the transfection mix was added to pre-washed cells. After 5 h, the transfection medium was replaced with DMEM supplemented with 10 % FBS. At 24 h post-transfection, images were acquired using a Zeiss Axio Cam Mrc-5 mounted onto a Zeiss Axioskop 40-fluorescent microscope.

### Virus generation

For influenza virus generation, BHK-21, CHO, or MDCK cells grown in 6-well plates were transfected with plasmid DNA as described above. After 5 h of incubation, the transfection medium was replaced with 2.5 mL of Opti-MEM containing penicillin and streptomycin. At 24 h post-transfection, the medium was replaced with 3 mL of Opti-MEM containing 0.2 μg/mL TPCK-trypsin, 0.3 % BSA, penicillin, and streptomycin. At three to six days post-transfection, cell supernates were titrated on MDCK cell monolayers to estimate influenza virus titers.
